# Simultaneous Recruitment of Stem Cells and Chondrocytes Induced by a Functionalized Self-Assembling Peptide Hydrogel Improves Endogenous Cartilage Regeneration

**DOI:** 10.3389/fcell.2020.00864

**Published:** 2020-08-27

**Authors:** Xiao Lv, Caixia Sun, Binwu Hu, Songfeng Chen, Zhe Wang, Qiang Wu, Kun Fu, Zhidao Xia, Zengwu Shao, Baichuan Wang

**Affiliations:** ^1^Department of Orthopaedics, Union Hospital, Tongji Medical College, Huazhong University of Science and Technology, Wuhan, China; ^2^Department of Gynecology, General Hospital of the Yangtze River Shipping, Wuhan, China; ^3^Department of Orthopaedics, The First Affiliated Hospital of Zhengzhou University, Zhengzhou, China; ^4^Department of Orthopaedics, The First Affiliated Hospital of Hainan Medical University, Hainan, China; ^5^Centre for Nanohealth, ILS2, Swansea University Medical School, Swansea, United Kingdom

**Keywords:** osteochondral defect, self-assembling peptide, chondrocyte recruitment, stem cell recruitment, cartilage regeneration

## Abstract

The goal of treating articular cartilage (AC) injury is to regenerate cartilage tissue and to integrate the neo-cartilage with surrounding host cartilage. However, most current studies tend to focus on engineering cartilage; interface integration has been somewhat neglected. An endogenous regenerative strategy that simultaneously increases the recruitment of bone marrow mesenchymal stem cells (BMSCs) and chondrocytes may improve interface integration and cartilage regeneration. In this study, a novel functionalized self-assembling peptide hydrogel (KLD-12/KLD-12-LPP, KLPP) containing link protein N-peptide (LPP) was designed to optimize cartilage repair. KLPP hydrogel was characterized using transmission electron microscopy (TEM) and rheometry. KLPP hydrogel shared a similar microstructure to KLD-12 hydrogel which possesses a nanostructure with a fiber diameter of 25–35 nm. *In vitro* experiments showed that KLPP hydrogel had little cytotoxicity, and significantly induced chondrocyte migration and increased BMSC migration compared to KLD-12 hydrogel. *In vivo* results showed that defects treated with KLPP hydrogel had higher overall International Cartilage Repair Society (ICRS) scores, Safranin-O staining scores and cumulative histology scores than untreated defects or defects treated with KLD-12 hydrogel, although defects treated with KLD-12 and KLPP hydrogels received similar type II collagen immunostaining scores. All these findings indicated that the simple injectable functionalized self-assembling peptide hydrogel KLPP facilitated simultaneous recruitment of endogenous chondrocytes and BMSCs to promote interface integration and improve cartilage regeneration, holding great potential as a one-step surgery strategy for endogenous cartilage repair.

## Introduction

Articular cartilage (AC) defects commonly occur as a result of either trauma or degenerative disease. Due to its avascular nature, damaged AC has a limited intrinsic capacity for self-repair. For decades, many exogenous cell-based methods have been used to repair and regenerate damaged AC in both experimental models and clinical patients, and acceptable outcomes have been obtained (Nixon et al., 2017; McIntyre et al., 2018; Otto et al., 2018). However, these methods are usually associated with complicated procedures, donor-site morbidities and less controllable regulation during *ex vivo* cell expansion (Malda et al., 2003; Saei et al., 2017; Ozucer et al., 2018).

By bypassing the aforementioned disadvantages, *in situ* cartilage regeneration by recruiting endogenous stem cells (especially bone marrow mesenchymal stem cells, BMSCs) into the damaged sites might be a promising alternative. Excitingly, many approaches based on this concept, such as bone marrow stimulation and the use of inductive bioscaffold alone or in combination with chemoattractants, have been shown to largely regenerate damaged cartilage tissue (Williams and Harnly, 2007; Dai et al., 2016; Chen et al., 2019). However, in most studies, the integration of neo-cartilage with the surrounding host cartilage is poor or problematic (Poole, 2003). The reason for this failure might be attributed to the weakness or even lack of chondrocyte migration (Lu et al., 2013). In addition, studies showed that chondrocytes can promote chondrogenic differentiation of BMSCs, and prevent their hypertrophic differentiation (Fischer et al., 2010; Kim et al., 2019). Thus, an endogenous regenerative strategy that simultaneously increases recruitment of stem cells and chondrocytes may promote interface integration and improve cartilage regeneration.

Chemoattractants, such as growth factors and chemokines, play crucial roles *in situ* cell recruitment (Zhang et al., 2020). They can be delivered into local defect areas by being simply injected alone or loaded within scaffold matrix (Chuma et al., 2004; Malaeb et al., 2019). However, the use of these bioactive factors may result in some undesirable side-effects (van Beuningen et al., 2000; Miller et al., 2010). Several recent studies have shown that a number of short peptides exhibit similar biofunctions as growth factors/chemokines while avoiding their potential side-effects (Webber et al., 2010; Jayasuriya et al., 2016). These short peptides can be conjugated to the C-terminus of the self-assembling peptide sequences RADA-16 or KLD-12 to develop new functionalized self-assembling peptides (Wang et al., 2012; Liu et al., 2015). Such peptides can undergo spontaneous self-assembly to form nanofiber hydrogels. Importantly, peptide self-assembly does not eliminate the biofunctions of the short peptide itself. Recently, some designed functionalized self-assembling peptide hydrogels have been used for cartilage defect repair (Li et al., 2017; Sun et al., 2018). However, these designs mainly focused on cartilage regeneration by recruiting stem cells, the integration of neo-cartilage with host cartilage was somewhat neglected.

**FIGURE 1:**
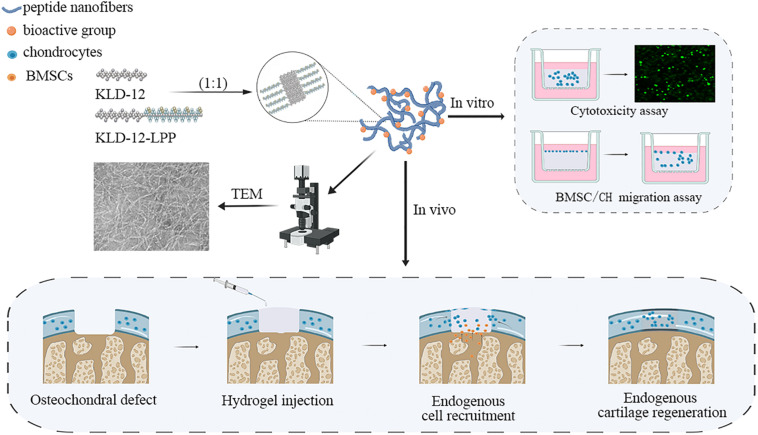
Schematic illustration of the overall research design. TEM, Transmission electron microscopy; BMSC, Bone marrow mesenchymal stem cells; CH, chondrocyte.

Link protein N-terminal peptide (LPP), as one of the link protein degradation products, plays an important role in regulating the proliferation and extracellular matrix (ECM) synthesis of chondrocytes (Liu et al., 2000; He et al., 2018). In addition, recent studies showed that LPP can also induce directional migration of nucleus pulposus cells (NPCs) and cartilage-derived stem cells (CSCs) (Wang et al., 2012; He et al., 2018). In this study, we used LPP to enrich KLD-12 to design a new functionalized peptide, KLD-12-LPP. A novel functionalized peptide hydrogel KLPP was formed by mixing peptide solutions of KLD-12 and KLD-12-LPP at a ratio of 1:1. We hypothesized that KLPP hydrogel could simultaneously increase the recruitment of endogenous BMSCs and chondrocytes to promote interface integration and improve cartilage regeneration. In the present study, KLPP hydrogel was characterized using transmission electron microscopy (TEM) and rheometry. We evaluated the effect of KLPP hydrogel on the migration of chondrocytes and BMSCs *in vitro*, while *in vivo* experiments were performed to test the ability of KLPP hydrogel to improve interface integration and cartilage regeneration in a rabbit osteochondral defect model. The overall research design is illustrated in Figure 1.

## Materials and Methods

### Peptide Synthesis and Self-Assembly

**FIGURE 2:**
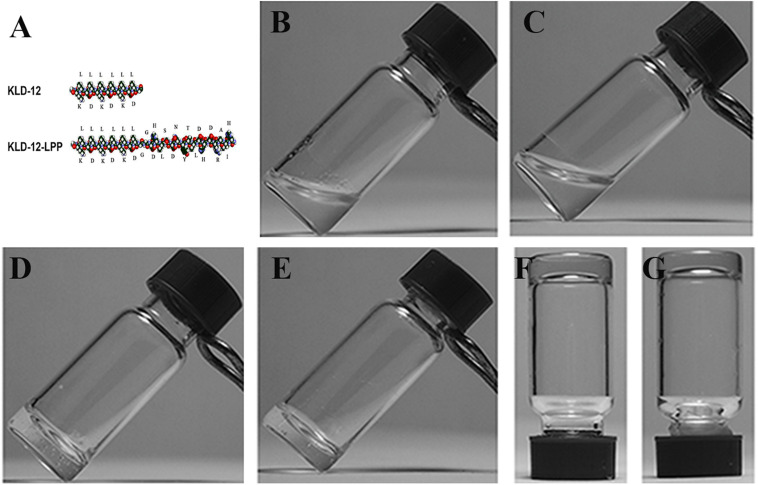
**(A)** Molecular models of KLD-12 and KLD-12-LPP peptides. Peptide solutions after sonication: **(B)** KLD-12 solution and **(C)** KLD-12-LPP/KLD-12 mixed solution. Peptide hydrogels: **(D)** KLD-12 hydrogel and **(E)** KLD-12/KLD-12-LPP (KLPP) hydrogel. The gel strength of the hydrogels was enhanced by addition of CaCl2 solution. **(F)** KLD-12 hydrogel and **(G)** KLPP hydrogel.

Self-assembling peptide KLD-12 with the sequence Ac-(KLDL)_3_-CONH2 and the new designed functionalized peptide KLD-12-LPP with the sequence Ac-(KLDL)_3_-GG-DHLSDNYTLDHDRAIH-CONH2 were custom-synthesized (GL Biochem Ltd., Shanghai, China). Purity and identification of the peptides were confirmed by high-performance liquid chromatography (HPLC) and mass spectrometry (MS). The molecular models of peptides KLD-12 and KLD-12-LPP are shown in Figure 2A.

The two peptide powders were dissolved in 10% (w/v) sterile sucrose solution at a peptide concentration of 1% (10 mg/mL), and sonicated for 30 min (Frequency: 40 kHz, DSA50-GL1, DESEN, China). KLD-12-LPP peptide solution was then mixed with KLD-12 peptide solution at a ratio of 1:1 (1%). KLD-12 solution and KLD-12/KLD-12-LPP mixed solution were allowed to stand for 30 min for complete gelation. In addition, CaCl2 (0.1 mol/L) solution was added to further improve and enhance peptide self-assembly.

### Microstructural Study

Transmission electron microscopy was used to identify the microstructure of KLD-12 and KLPP hydrogels. Peptide solution was diluted to a working concentration of 0.01% using phosphate-buffered saline (PBS), and then dropped onto freshly glow-discharged carbon-coated copper grids. Excess solution was blotted off with a filter paper, and samples were negatively stained with 1% uranyl acetate (pH 4.2), and then air-dried overnight. Peptide samples were observed by TEM (HT7700, Hitachi, Tokyo, Japan) at 80 kV, and images were acquired and analyzed using Digital Micrograph software (Gatan, Inc., Pleasanton, CA, United States).

### Rheological Measurement

The viscoelastic behaviors of KLD-12 and KLPP hydrogels were studied using an AR2000 rheometer (TA Instruments, New Castle, DE, United States). After sonication for 30 min, 100 μL of peptide hydrogel was loaded onto a parallel plate (diameter: 20 mm), and allowed to stand for 30 min for gelation. The frequency sweep tests of storage modulus (G′) and loss modulus (G″) were measured at 25°C with parameters of strain 0.5% and a frequency range of 0.01–100 rad/s.

### Isolation of Chondrocytes and BMSCs

All animal experiments were approved by the animal ethical committee of our university. Rabbit chondrocytes and BMSCs were isolated and cultured as described by Mohan et al. (2018) and Wang et al. (2014), respectively. In brief, cartilage was aseptically harvested from rabbit knee joint and digested with 0.25% type II collagenase (Sigma, St. Louis, MO, United States) to isolate chondrocytes. BMSCs were harvested by density gradient centrifugation and adherence methods. Second generation chondrocytes and third generation BMSCs were used for subsequent experiments.

### Hydrogel Preparation

To reduce potential interference of manual operation with the hydrogel scaffold when cell culture medium was changed, transwell plates (Sigma, St. Louis, MO, United States) were used in our *in vitro* experiments. After sonication for 30 min, 60 μL of hydrogel (KLD-12 or KLPP) was added to a cell culture insert (insert diameter: 6.5 mm; BD Biosciences, Franklin Lakes, NJ, United States), and formed a layer approximately 1–1.5 mm in thickness. Dulbecco’s modified Eagle’s medium (DMEM, Gibco Invitrogen, Carlsbad, CA, United States) was gently added to each insert to enhance peptide self-assembly. After gelation, the medium was changed every 4 h over a 12 h period to equilibrate the hydrogel to physiological pH.

### Cytotoxicity Assay

In our experiments, chondrocytes were chosen to evaluate the cytotoxicity of KLPP hydrogel. A suspension of chondrocytes was added into cell culture inserts and mixed with each peptide hydrogel (5 × 10^4^ cells/insert), and culture medium was gently added. After 3 or 7 days of culture, cell/hydrogel complexes were rinsed twice with PBS and stained with Calcein-AM (CAM; 5 μg/mL; Sigma) and Propidium Iodide (PI; 5 μg/mL; Sigma) for 30 min, then rinsed three times with PBS. Live and dead cells were counted in five randomly-selected non-overlapping fields.

### Cell Migration Assay

To observe whether KLPP hydrogel could induce spontaneous chondrocyte or BMSC migration, cells (3 × 10^4^ cells/insert) were seeded onto the surface of KLD-12 or KLPP hydrogel. After 7 days of culture, cell/hydrogel complexes were stained with CAM (5 μg/mL; Sigma) for 30 min, and then gently rinsed three times with PBS. Images were captured by laser scanning confocal microscopy (LSCM; Zeiss 710, Carl Zeiss Microscopy GmbH, Jena, Germany). All the experiments were repeated at least three times.

### *In vivo* Study

#### Osteochondral Defect Creation and Hydrogel Injection

Twenty-one New Zealand white rabbits (average body weight, 4.5 kg; average age: 11.2 months) were selected for animal experiments. Rabbits were anesthetized using pentobarbital (0.2 g/mL), and their knees were shaved and sterilized. The knee joint was exposed via an anteromedial parapatellar incision. An osteochondral defect (3.5 mm diameter × 2 mm deep) was created in the central region of the femoral trochlear groove.

Hemostasis was performed with medical gauze and gelatin sponge before use of the peptide hydrogel, which was delivered as a liquid. Defects were filled fully as dictated by group assignment. The experiment was divided into three groups: group 1 (untreated), group 2 (treated with KLD-12 hydrogel), and group 3 (treated with KLPP hydrogel). In order to prevent/reduce the loss of hydrogel implants, the newly-formed hydrogels were allowed to stand for 15–30 min for complete gelation, and the knees were fixed for 1 week after surgery. Rabbits were euthanized at 12 weeks, and knee joints were harvested for subsequent analyses. One rabbit in group 2 died during the study due to post-operative infection and was not included in the analyses.

#### Macroscopic Evaluation of Repaired Cartilage

The macroscopic outcome of repaired cartilage was assessed blindly using the International Cartilage Repair Society (ICRS) macroscopic scoring system with a maximum overall repair score of 12 and a higher score indicating a repair more like normal cartilage. The parameters included: (a) level with surrounding cartilage, (b) integration to border zone, and (c) surface macroscopic appearance (van den Borne et al., 2007).

#### Histological and Immunohistochemical Evaluations of Repaired Cartilage

The repaired tissue along with surrounding native tissue was fixed in neutral-buffered 10% formalin. The fixed tissue was decalcified, embedded in paraffin, and sectioned at 4 μm. Sections were stained with Hematoxylin and Eosin (H&E) (PHYGENE, Fujian, China) and Safranin-O/Fast green (SOFG) (Sigma) for histological analyses, and rabbit anti-type II collagen (1:800, Proteintech, 28459-1-AP, Wuhan, China) for immunohistochemical analyses. The staining procedures were performed according to the manufacturer’s instructions. The neo-cartilage tissues were assessed using a modified O’Driscoll histological scoring system based on sections with H&E and SOFG staining (Miller et al., 2010; Rutgers et al., 2010) and Miller’s immunohistochemical scoring system based on sections with type II collagen staining (Miller et al., 2010).

### Statistical Analysis

Statistical analysis was performed using IBM SPSS software 26.0 (IBM Corp., Armonk, NY, United States). Differences in the number of live cells between KLD-12 and KLPP groups were determined using the independent-group *t*-test. The Kruskal-Wallis test was used to compare macroscopic scores, histological scores and immunohistochemical scores between groups. Differences were considered as statistically significant when *P* < 0.05.

## Results

### Synthesis and Peptide Self-Assembly

Successful synthesis and purification of peptides KLD-12 and KLD-12-LPP were confirmed by HPLC and MS. The molecular weight of KLD-12 was 1467.83 Da with a purity of 96.56%, while that of KLD-12-LPP was 3485.93 Da with a purity of 97.14%.

After dissolving in 10% sterile sucrose, KLD-12 formed a transparent viscous hydrogel, whereas KLD-12-LPP remained a non-viscous solution. However, a hydrogel was formed when KLD-12-LPP peptide solution was mixed with KLD-12 peptide solution at a ratio of 1:1. Moreover, the gel strength of the hydrogels was improved by addition of CaCl2 solution, which indicated that peptide self-assembly could be further enhanced by addition of divalent ions (Figure 2).

### Characterization of Peptide Hydrogels

**FIGURE 3:**
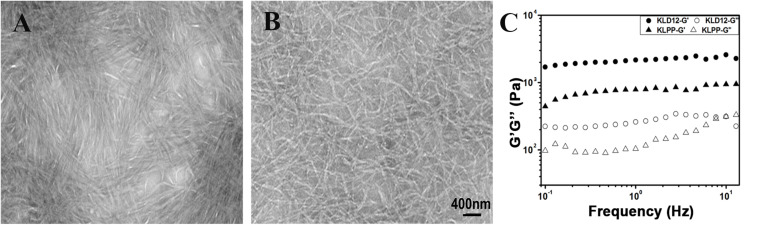
**(A,B)** Representative transmission electron microscopy images of panel **(A)** KLD-12 hydrogel and **(B)** KLPP hydrogel. **(C)** Rheological properties of KLD-12 and KLPP hydrogels.

The microstructure of peptide hydrogels was examined using TEM. As previously reported (Sun and Zheng, 2009), nanofibers were formed in KLD-12 with an average fiber diameter of approximately 25–35 nm. KLD-12-LPP alone did not form nanofibers. However, nanofibers were observed in KLPP, which appeared broadly similar to the structure of KLD-12 (Figures 3A,B).

The storage modulus (G′) and loss modulus (G″) respond to the elasticity and viscosity of materials, respectively. As shown in Figure 3C, the frequency sweep results showed that the values of G’ over the entire frequency range exceeded those of G′ in both KLD-12 and KLPP, which revealed the gel-like properties of the two peptide hydrogels.

### Cytotoxicity of KLPP Hydrogel

**FIGURE 4:**
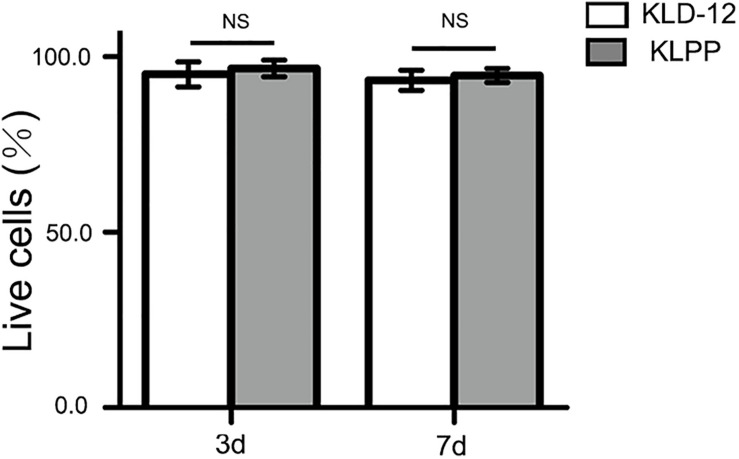
Quantification assay of cell survival rates. Cell viability = live cells/(live cells + dead cells) × 100%. There was no significant difference in cell survival rates between KLD-12 and KLPP hydrogels after 3 and 7 days of culture (*n* = 5, mean ± SD. KLD-12 vs. KLPP: 95.2 ± 3.6% vs. 96.5 ± 2.4% on day 3; 93.4 ± 2.9% vs. 94.5 ± 1.9% on day 7. NS: no significance, *P* > 0.05).

Live/dead staining was used to evaluate the viability of chondrocytes encapsulated in the peptide hydrogels. The live cells showed a green fluorescence, while the dead cells showed a red fluorescence. KLD-12 hydrogel, a good substrate for 3D cell culture, was used as control (Kisiday et al., 2002). As shown in Figure 4, the proportion of live cells encapsulated within both peptide hydrogels was more than 90% and there were no significant difference between groups (*P* > 0.05).

### Cell Migration Induced by KLPP Hydrogel

**FIGURE 5:**
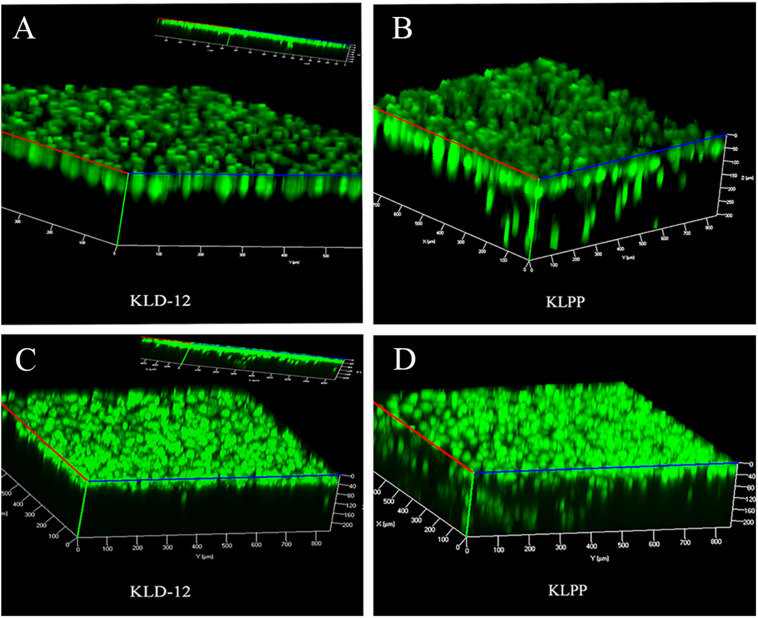
3D confocal images of chondrocyte **(A,B)** and BMSC **(C,D)** migration on day 7. **(A,C)** KLD-12 hydrogel, **(B,D)** KLPP hydrogel. KLPP hydrogel significantly induced chondrocyte migration and increased BMSC migration compared to KLD-12 hydrogel. 3D, three-dimensional; BMSC, Bone marrow mesenchymal stem cells.

To observe spontaneous cell migration, cells were seeded onto the surface of KLD-12 or KLPP hydrogel, and observed using LSCM after staining with CAM on day 7. Three-dimensional (3D) confocal images showed that most chondrocytes seeded onto the surface of KLPP hydrogel spontaneously migrated into the hydrogel (up to 300 μm), whereas almost all cells still remained on the surface of KLD-12 hydrogel. Unlike chondrocytes, BMSCs penetrated into both peptide hydrogels, but more migrated cells with greater migration distances were observed in KLPP hydrogel when compared with KLD-12 hydrogel (Figure 5).

### Gross Observation of Repaired Cartilage

**FIGURE 6:**
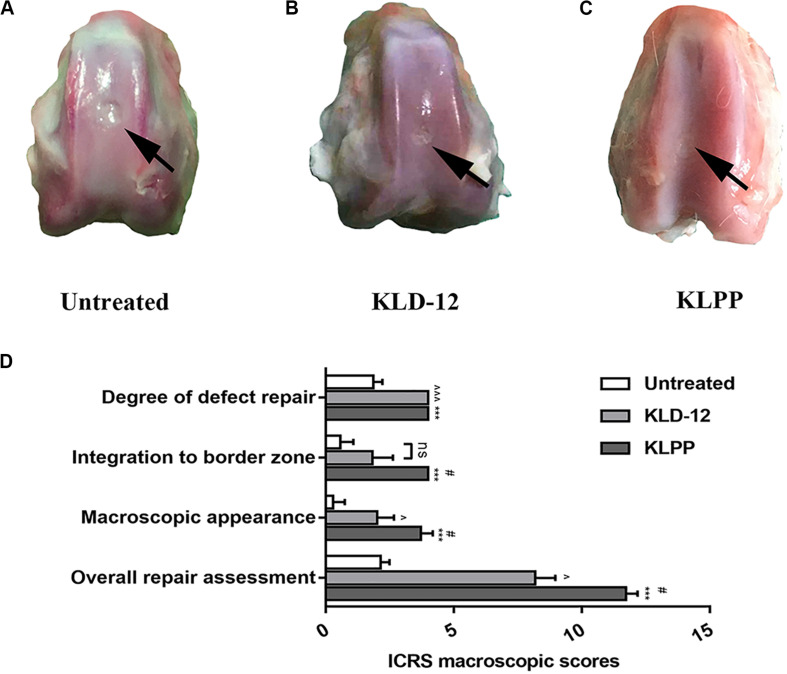
Gross necropsy photographs of untreated defects **(A)** and defects treated with KLD-12 hydrogel **(B)** and KLPP hydrogel **(C)**. **(D)** ICRS macroscopic scores at 12 weeks. (*n* = 3, mean ± SD.*, KLPP vs. Untreated, ***p < 0.001; #, KLPP vs. KLD-12, ^#^*p* < 0.05; ^∧^, KLD-12 vs. Untreated, ^∧^*p* < 0.05, ^∧∧∧^*p* < 0.001. ns: no significance).

Upon necropsy, incision areas and joints in all groups appeared normal by gross examination, and no significant inflammation was noted, indicating that there were no adverse immune reactions to these peptide hydrogels. In the untreated group (group 1), the defects became shallow and were filled with fibrous-like tissues. Satisfactory filling was observed in all defects treated with KLD-12 and KLPP hydrogels. Most of the repaired tissues from the KLD-12 group (group 2) did not integrate completely with the surrounding host cartilage, with small scattered fissures remaining. The repaired tissue from the KLPP group (group 3) was semi-transparent in appearance, similar to hyaline cartilage, and appeared to be well integrated with the surrounding host cartilage (Figures 6A–C).

The ICRS scoring system was used for macroscopic evaluation of repaired cartilage. Overall, treated defects received higher overall ICRS scores compared to untreated defects (Figure 6D). Group 3 had higher scores in terms of integration to the border zone (*P* = 0.024) and macroscopic appearance (*P* = 0.048), although no difference between the level of the defect surface compared to the surrounding cartilage was observed between groups 2 and 3.

### Histological and Immunohistochemical Evaluations of Repaired Cartilage

**FIGURE 7:**
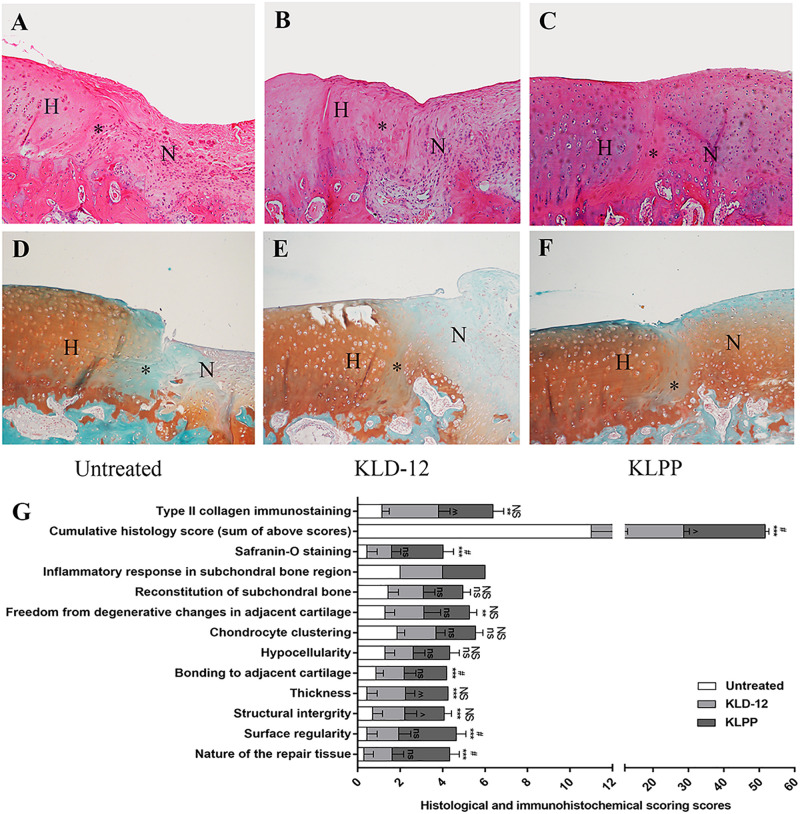
H&E staining **(A–C)** and Safranin-O/Fast green staining **(D,E)** of repaired cartilage. Images showing evaluation of untreated defects **(A,D)** and defects treated with KLD-12 hydrogel **(B,E)** and KLPP hydrogel **(C,F)**. (Magnification × 100). (H, host cartilage; N, neo-cartilage; *, the interface between neo-cartilage and host cartilage). **(G)** Histological and immunohistochemical scores at 12 weeks. (*n* = 3, mean ± SD. *, ns, KLPP vs. Untreated, *p < 0.05, **p < 0.01, ***p < 0.001; #, NS, KLPP vs. KLD-12, ^#^*p* < 0.05, ^##^*p* < 0.01, ^###^*p* < 0.001; ^∧^, ns, KLD-12 vs. Untreated, ^∧^*p* < 0.05, ^∧∧^*p* < 0.01. NS/ns: no significance).

Neo-cartilage tissues were further evaluated by histological and immunohistochemical staining (Figure 7). Histological evaluation based on H&E and SOFG staining showed that the KLPP group (group 3) had a higher cumulative score when compared with the untreated group (group 1) (*P* < 0.001) and the KLD-12 group (group 2) (*P* = 0.048). Defects treated with KLPP hydrogel received the highest score for the nature of the repaired tissue, indicating that it consisted mostly of hyaline cartilage; in contrast, scores of groups 1 and 2 ranged between 0 and 1, showing some fibrous tissue and mostly fibrocartilage. Compared with group 2, group 3 had a higher score for surface regularity, suggesting the presence of a smooth and intact cartilage surface. Group 3 also had the highest score for bonding to adjacent cartilage, showing good interface integration between the neo-cartilage and surrounding host cartilage, compared to groups 1 and 2. Repaired tissue in group 3 had significantly increased cellularity compared to groups 1 and 2, and the morphology of the replacement cells was similar to that of chondrocytes (Figure 7F). Reconstitution of subchondral bone was observed in all groups, but they were all below normal subchondral bone levels and there were no significant difference between groups.

As shown in Figures 7D–F, group 3 received significantly higher Safranin-O staining scores (moderate to normal staining), indicating more proteoglycan aggregation, compared to groups 1 and 2. For immunohistochemical analyses, groups 2 and 3 had higher type II collagen immunostaining scores compared to group 1, but there was no significant difference between groups 2 and 3.

## Discussion

Once damaged, AC has a very limited self-repair ability. Even small injuries would progress to larger and deeper lesions over time if left untreated, and eventually lead to degeneration and osteoarthritis of the whole joint (Davies-Tuck et al., 2008). The main objective of this study was to attempt to design a novel scaffold that could be used *in situ* cartilage regeneration. A new functionalized self-assembling peptide hydrogel, KLPP, was specially designed containing the bioactive motif LPP. KLPP hydrogel shared similar nanostructure and rheological properties to traditional KLD-12 hydrogel. However, compared with KLD-12 hydrogel, KLPP hydrogel significantly facilitated the infiltration of chondrocytes and BMSCs, and thus promoted interface integration and superior cartilage regeneration.

In recent years, *in situ* cartilage regeneration by recruiting endogenous stem cells has received more attention (Williams and Harnly, 2007; Dai et al., 2016; Chen et al., 2019). It utilizes the body’s own regeneration capacity to repair cartilage tissue while avoiding exogenous cell-associated limitations. Microfracture is a common surgical technique for AC repair, which employs the concept of BMSC recruitment to regenerate cartilage tissue (Williams and Harnly, 2007). However, the neo-tissues are mostly comparatively weak fibrous cartilage rather than hyaline cartilage. The reasons for this failure could be attributed to an inadequate number of recruited stem cells (Im, 2016). Thus, many strategies, such as application of chemoattractants or inductive bioscaffolds, alone and in combinations, have been used to improve and enhance the recruitment of endogenous stem cells, and regenerate cartilage tissue with some success (Chuma et al., 2004; Dai et al., 2016; Chen et al., 2019). However, most of these neo-cartilage tissues would deteriorate over time as a result of the lack of lateral integration between engineered neo-cartilage and host cartilage (Horas et al., 2003).

Various studies have demonstrated that chondrocyte migration is an important contributor to the formation of an integrative interface (Pabbruwe et al., 2009; Lu et al., 2013). Recruited chondrocytes at the interface sites can drive ECM remodeling, improving tissue integration of the interface (Pabbruwe et al., 2009). A growing body of evidence has shown that chondrocyte migration can be improved and accelerated by creating an appropriate matrix microenvironment. Levinson et al. (2019) embedded cartilage particles into fibrin and collagen gels, and observed significant chondrocyte outgrowth after 28 days in culture. Ng et al. (2012) reported that a macro-porous polyvinyl alcohol (PVA) scaffold facilitated chondrocyte migration from host cartilage into scaffold and improved interface integration in an *in vitro* cartilage defect model. In addition to improving interface integration, infiltrated chondrocytes also promote chondrogenic differentiation of recruited BMSCs (Kim et al., 2019). Therefore, a functionalized scaffolding system that can effectively induce simultaneous migration of BMSCs and chondrocytes might promote interface integration and improve endogenous cartilage regeneration.

Self-assembling peptide hydrogel scaffolds possess a nanostructure with fiber diameter of 10–50 nm, pore size of 5–200 nm, and water content of more than 99%, which ideally mimics the nanostructure of the natural ECM (Kisiday et al., 2002; Wang et al., 2012). Studies showed that such architecture itself facilitates cell migration and chondrogenic differentiation of BMSCs (Miller et al., 2010). More importantly, self-assembling peptide hydrogels (such as RADA-16 and KLD-12) can be functionalized by adding bioactive motifs at the C-terminus of the self-assembling peptide sequences (Wang et al., 2012; Liu et al., 2015). Such a functionalized peptide hydrogel might provide a more suitable matrix environment for cell migration and differentiation. As mentioned above, LPP plays an important role in regulating biological behaviors of chondrocytes, NPCs and CSCs (Liu et al., 2000; Wang et al., 2012; He et al., 2018). In the present study, we specially selected LPP to enrich KLD-12 to construct a novel functionalized peptide hydrogel scaffold: KLPP, and investigated its effects on the migration of chondrocytes and BMSCs *in vitro* and interface integration and cartilage regeneration *in vivo*.

Our results showed that the functionalized peptide KLD-12-LPP alone did not form hydrogel. One possible reason is that the relatively long LPP motif interferes with inter-molecular interactions to affect peptide self-assembly (Tao et al., 2015). However, a stable functionalized hydrogel consisting of nanofiber networks was formed when the peptide solutions of KLD-12 and KLD-12-LPP were mixed at a ratio of 1:1 (Figures 2, 3), which is similar to many previous reports (Wang et al., 2014; Tao et al., 2015). As shown in Figure 4, KLPP hydrogel shared similar cytotoxicity to KLD-12 hydrogel. *In vitro* cell migration experiments, most chondrocytes spontaneously migrated into KLPP hydrogel, whereas almost all cells still remained on the surface of KLD-12 hydrogel. These exciting results might be attributed to the LPP receptor on chondrocytes, BMP-RII (Wang et al., 2013). BMSCs, unlike chondrocytes, infiltrated into both KLD-12 and KLPP hydrogels. However, larger numbers of cells which migrated farther into the hydrogel were observed in KLPP hydrogel compared to KLD-12 hydrogel (Figure 5). The different migration effects might be associated with the different migration capabilities of chondrocytes and BMSCs or the different microenvironments provided by these two peptide hydrogels.

Rabbit osteochondral defects are known to have a high endogenous self-repair potential. However, studies have shown that a large osteochondral defect (≥3 mm in diameter) in a mature rabbit does not self-heal (Qiu et al., 2003). In this study, a 3.5-mm diameter osteochondral defect was created in rabbit trochlea, and our results showed that all untreated defects remained unhealed and were filled with mostly fibrous tissue or fibrous cartilage. Moreover, the presence of a 2 mm-deep defect allowed BMSC migration from underlying bone marrow into cartilage defects, similar to the effects of the microfracture technique and abrasion arthroplasty (Williams and Harnly, 2007; Miller et al., 2010). Our *in vivo* results showed that defects treated with KLPP hydrogel had higher overall ICRS scores, Safranin-O staining scores and histological scores, indicating better interface integration and cartilage regeneration, compared to untreated defects or defects treated with KLD-12 hydrogel, although defects treated with KLD-12 and KLPP hydrogels received similar type II collagen immunostaining scores (Figures 6, 7). The satisfactory healing results might be attributed to increased recruitment of chondrocytes and BMSCs induced by KLPP hydrogel. Moreover, the positive response might also partly be caused by the recruitment of other stem cells, such as CSCs, synovium-derived stem cells and articular fat pad-derived stem cells, in spite of very limited cell numbers (Im, 2016; Zhang et al., 2020). Of note, although reconstruction of subchondral bone was observed, all groups were still below normal subchondral bone levels. In clinical practice, KLPP hydrogel, combined with microfracture, can be delivered into cartilage defects for endogenous cartilage regeneration.

Considering the above, the functionalized peptide hydrogel KLPP might have great potential in endogenous cartilage regeneration. Firstly, the peptide solution can be injected arthroscopically to fill an irregular defect *in situ* and rapidly self-assemble into a stable hydrogel once it is in contact with damaged tissues. Secondly, these hydrogels completely synthetic and can be bio-degraded in the body without inducing an immune response. *In vivo* experiments, no significant inflammatory response was noted in any of the groups. Finally and most importantly, the functionalized peptide hydrogel possesses ECM-like nanostructure with a high density of the LPP bioactive group, which can simultaneously induce the migration of endogenous stem cells and chondrocytes and provide an ideal matrix microenvironment for these infiltrating cells. Moreover, they can also work as a controlled release system for drug delivery (such as anti-inflammatory drugs) to further improve cartilage regeneration (Miller et al., 2010; Tao et al., 2015).

Admittedly, our study has some limitations. One main limitation of this study is that the experimental design is more functional rather than molecular. An accurate evaluation at the molecular level may be helpful to further validate the effect of the novel functionalized KLPP hydrogel. Another limitation of this study is that the rabbit osteochondral defect model suffers from relatively thin cartilage and a high endogenous self-healing potential. A larger animal model with a standard osteochondral defect is needed to further confirm our experimental results.

## Conclusion

In this study, we designed a simple injectable functionalized peptide hydrogel KLPP for promoting interface integration and improving endogenous cartilage regeneration. KLPP hydrogel has the ability to induce and enhance the migration of chondrocytes and BMSCs in an *in vitro* 3D culture system. *In vivo* results showed that KLPP hydrogel induced superior hyaline-like cartilage repair and improved interface integration. Hence, the functionalized KLPP hydrogel would be a promising candidate for endogenous cartilage regeneration without the employment of any extraneous cells or chemoattractants.

## Data Availability Statement

The raw data supporting the conclusions of this article will be made available by the authors, without undue reservation.

## Ethics Statement

The animal study was reviewed and approved by Ethics Committee of Tongji Medical College, Huazhong University of Science and Technology.

## Author Contributions

BW and ZS conceived and designed the study. XL, CS, BH, SC, and ZW acquired the data. XL, CS, SC, BH, KF, and QW analyzed and interpreted the data. XL, BW, and ZX drafted the manuscript. ZX, BW, and ZS contributed to the critical revision of the manuscript for important intellectual content. All authors approved the final version of the submitted manuscript.

## Conflict of Interest

The authors declare that the research was conducted in the absence of any commercial or financial relationships that could be construed as a potential conflict of interest.
